# Tumour-to-tumour metastasis: head and neck squamous cell carcinoma to enchondroma

**DOI:** 10.1007/s00256-026-05225-z

**Published:** 2026-05-05

**Authors:** Robert Henderson, Daniel Wong, Richard Carey-Smith, Rajesh Botchu

**Affiliations:** 1 Department of Musculoskeletal Radiology, Royal Orthopaedic Hospital NHS Foundation Trust, Birmingham, UK; 2 Joint Department of Medical Imaging, Musculoskeletal Division, University Health Network, Toronto, Canada; 3 Department of Medical Imaging, Perth Children’s Hospital, Perth, Western Australia Australia; 4 Anatomical Pathology, PathWest Laboratory Medicine, Perth, WA Australia; 5 Clinical Lead State Sarcoma Service, Department of Orthopaedics, Sir Charles Gairdner Hospital, Perth, Western Australia Australia; 6 University of Western Australia, Crawley, WA Australia; 7 Department of Orthopaedics, Perth Children’s Hospital, Perth, Western Australia Australia; 8 Orthopaedic and Sports Medicine Centre, West Perth, Perth, Western Australia Australia

**Keywords:** Tumour-to-tumour, Metastasis, Collision, Enchondroma, Cancer, Carcinoma

## Abstract

Metastatic disease is a common occurrence in malignancy with certain cancers recognised to have a high incidence of metastases, particularly the liver, lungs, and bones. Tumour-to-tumour metastases are an exceptionally rare occurrence, where a distant malignancy metastasises to a secondary distinct tumour. Breast and lung carcinomas are the more commonly reported origin sites, whilst renal cell carcinomas and meningiomas are the most reported recipient malignant and benign tumours respectively. In this article, we describe an unusual case of a metastatic head and neck squamous cell carcinoma (HNSCC) complicated by tumour-to-tumour metastasis to a benign intramedullary cartilage lesion in the distal femur.

## Introduction

The typical pattern of metastatic disease involves indiscriminate systemic spread of cancer throughout the body, with preferential dissemination through various organs and lymph nodes. Whilst multiple primary malignancies may develop and coexist within an individual, tumour-to-tumour metastasis (TTM) characterises the rare and unusual circumstance of metastatic spread from a ‘donor’ tumour to a distinct secondary benign or malignant ‘recipient’ tumour [1–4].

HNSCC is a group of malignancies derived from the nasal, oral, pharyngeal, laryngeal and salivary glands of the head and neck region. HNSCC is the seventh most common cancer worldwide, accounting for an annual mortality of 450,000 and approximately 4.5% of cancer deaths [5–7].There are an estimated 54,000 new cases of HNSCC diagnosed in the United States in 2022 with approximately 11,230 deaths—which accounted for roughly 2% of all cancer deaths [5]. Despite the significant volume of metastatic disease associated with HNSCC, with 15% of patients already stage IV at diagnosis (where metastases were identified at their initial staging scan) [Barsouk 2023] [4], there are few instances of TTM reported in the literature [8–13].

Whilst a total of six HNSCC cases of TTM are documented to cervical nodal B-cell [9] and mantle cell [13] lymphoma, meningiomas [9, 10], and renal cell carcinomas [11, 12] have been reported previously, we report a case where HNSCC metastasised to an enchondroma, which at first assessment masqueraded as dedifferentiated chondrosarcoma.

## Case Report

We present the case report of a 56-year-old male currently undergoing treatment for metastatic HNSCC, having recently completed radiotherapy treatment for recurrent lung metastases. At this time his disease status was considered stable and he was entering a program of disease surveillance.

**Fig. 1:**
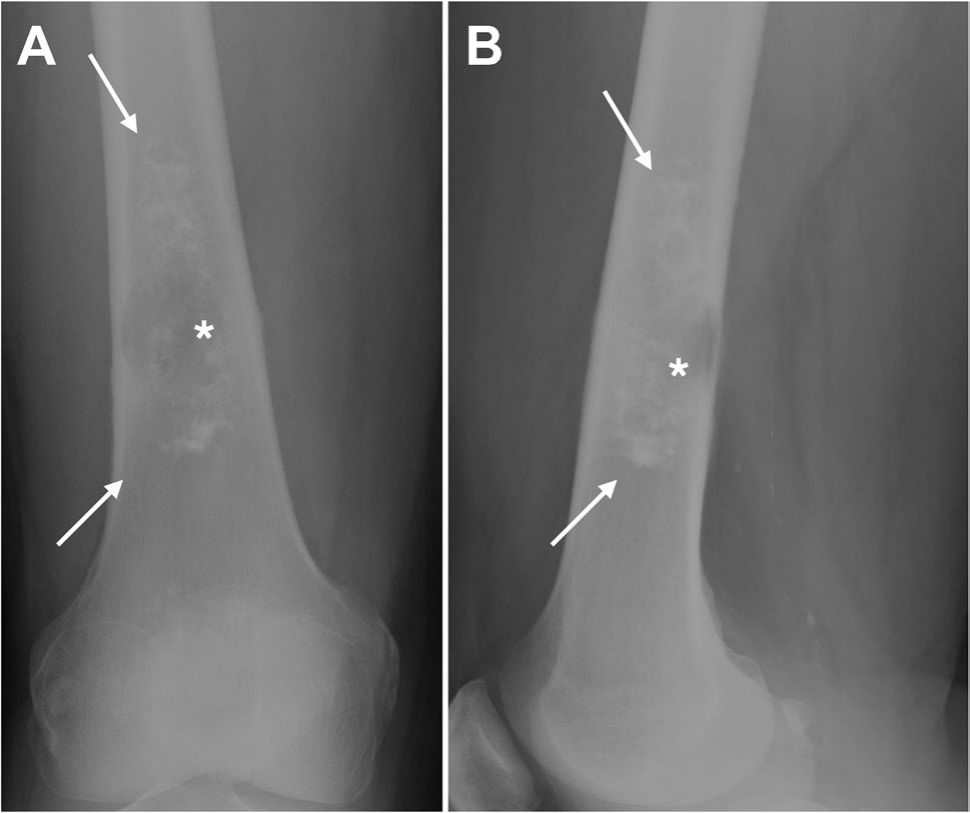
Initial [A] anteroposterior and [B] lateral radiographs of the distal femur demonstrating the biphasic lesion in the distal femoral shaft characterised by an elongated ovoid intramedullary mass with radiodense chondroid matrix mineralisation outlined at its craniocaudal margins (→), with central lytic component posteriorly (*) localised deep endosteal scalloping/periosteal reaction

Following complaints of slowly worsening persistent right hip and mid-thigh pain, a radiograph was initially obtained. The patient had previously undergone an uncomplicated right total hip arthroplasty. The femur radiograph [Fig. 1A, B] demonstrates an intramedullary lesion in the distal femoral shaft measuring 92 mm craniocaudal with characteristics of a typical chondroid lesion with an additional aggressive lytic component posteriorly with deep endosteal scalloping, periosteal reaction and focal cortical breach. A working diagnosis of dedifferentiated chondrosarcoma arising from the underlying enchondroma was suspected.

**Fig. 2:**
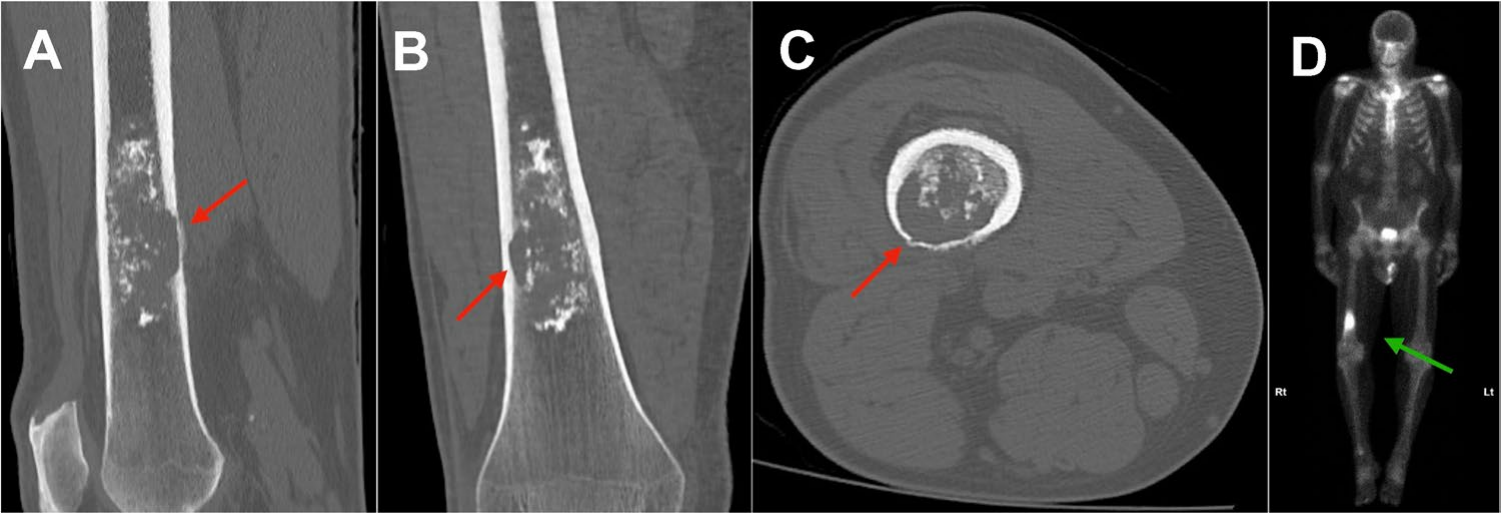
(**A–D**) CT demonstrates the biphasic pattern of the mass with the characteristic ‘ring and arc’ pattern of cartilage matrix mineralisation of the intramedullary lesion, and focal lytic areas with deep endosteal scalloping posteriorly (red→); bone scan [D] demonstrating uniformly avid tracer uptake in the solitary bone lesion in the distal femoral shaft (green→) in comparison to the anterior iliac crest

Further investigation with unenhanced CT [Fig. 2A–C] better characterised the chondroid matrix mineralisation typical of a benign intramedullary enchondroma, whereas the localised destructive lytic component eroding the cortex with focal full-thickness breach and periosteal reaction supported the concerns for in situ malignant transformation of the underlying enchondroma. Given the concurrent metastatic head and neck malignancy, a nuclear medicine bone scan SPECT was ordered for both complimentary assessment and exclusion of additional lesions or metastatic bone disease. Only the solitary femoral lesion demonstrated abnormal tracer uptake [Fig. 2D].

**Fig. 3:**
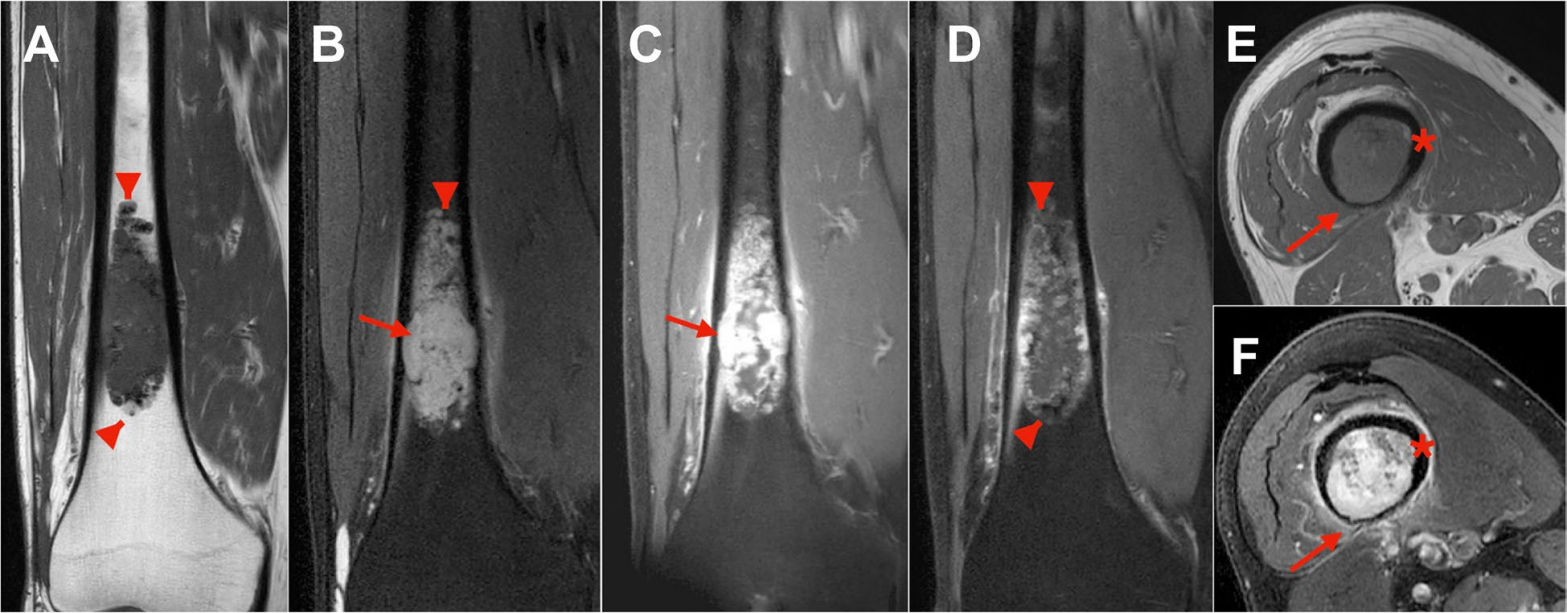
Further correlation between series of coronal T1-weighted (**A**), T2 fat-saturated (FS) and (**B**), coronal T1 FS post-contrast (**C, D**), plus axial T1 (**E**), and axial T1 FS post-contrast (**F**) demonstrating the difference in signal intensity between the enchondroma (➤) with mild peripheral enhancement, and HNSCC TTM (→) with a solid nodular enhancement pattern. The axial images demonstrate the deep endosteal scalloping from the TTM (→)  not seen by the enchondroma (*)

MRI showed the biphasic imaging characteristics and enhancement pattern of the mass [Fig. 3] with the low-signal chondroid mineralisation was visualised in the larger cartilage lesion with mild peripheral enhancement. The aggressive posterior component demonstrates a homogeneous solid T1 intermediate signal with uniform enhancement, endosteal erosion and irregular circumferential enhancing periosteal oedema, corresponding to the solid lytic destructive component on CT [Fig. 3D-–F].

Multimodality imaging characteristics and biphasic morphology were altogether supportive of the working diagnosis of dedifferentiated chondrosarcoma arising from an underlying enchondroma. Following discussion at the State Sarcoma Service Multidisciplinary Meeting, the consensus decision was for en bloc oncological resection of the mass with surgical reconstruction of the distal femur with hinged arthroplastic endoprosthesis.

**Fig. 4:**
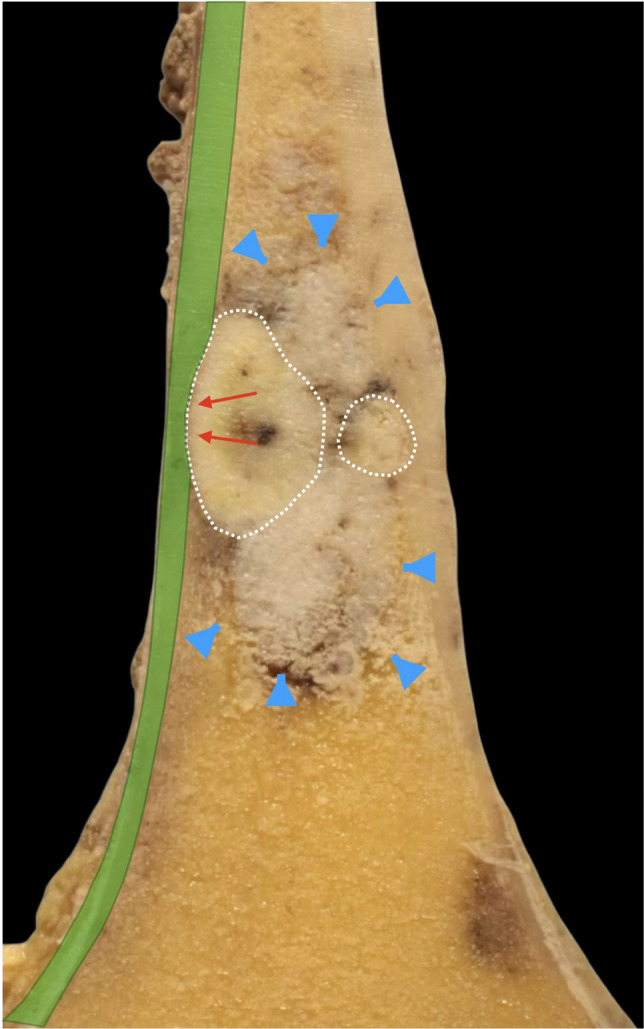
Macroscopic post-resection specimen of the distal femur demonstrating the biphasic components of the lesion; the background chalky white enchondroma at the superior and inferior margins (blue ➤) with the well-defined lobulated areas of cheesy pale-yellow TTM lesions (white outline) and areas of shallow scalloping (red →) of the endosteum (green)

En bloc surgical resection identified the malignant squamous cell carcinoma tumour-to-tumour metastasis to the benign intramedullary enchondroma. On cut section [Fig. 4], there was the larger pale glistening appearance of the benign cartilage lesion (*) with the smaller peripheral solid areas of expanding creamy/pale yellow tissue (arrows) associated with the endosteal erosion and cortical thinning indicating the areas of metastatic SCC.

Radiopathological correlation demonstrating the different components of the mass across multiple different modalities [Figs. 1, 2, 3, and 4].

**Fig. 5:**
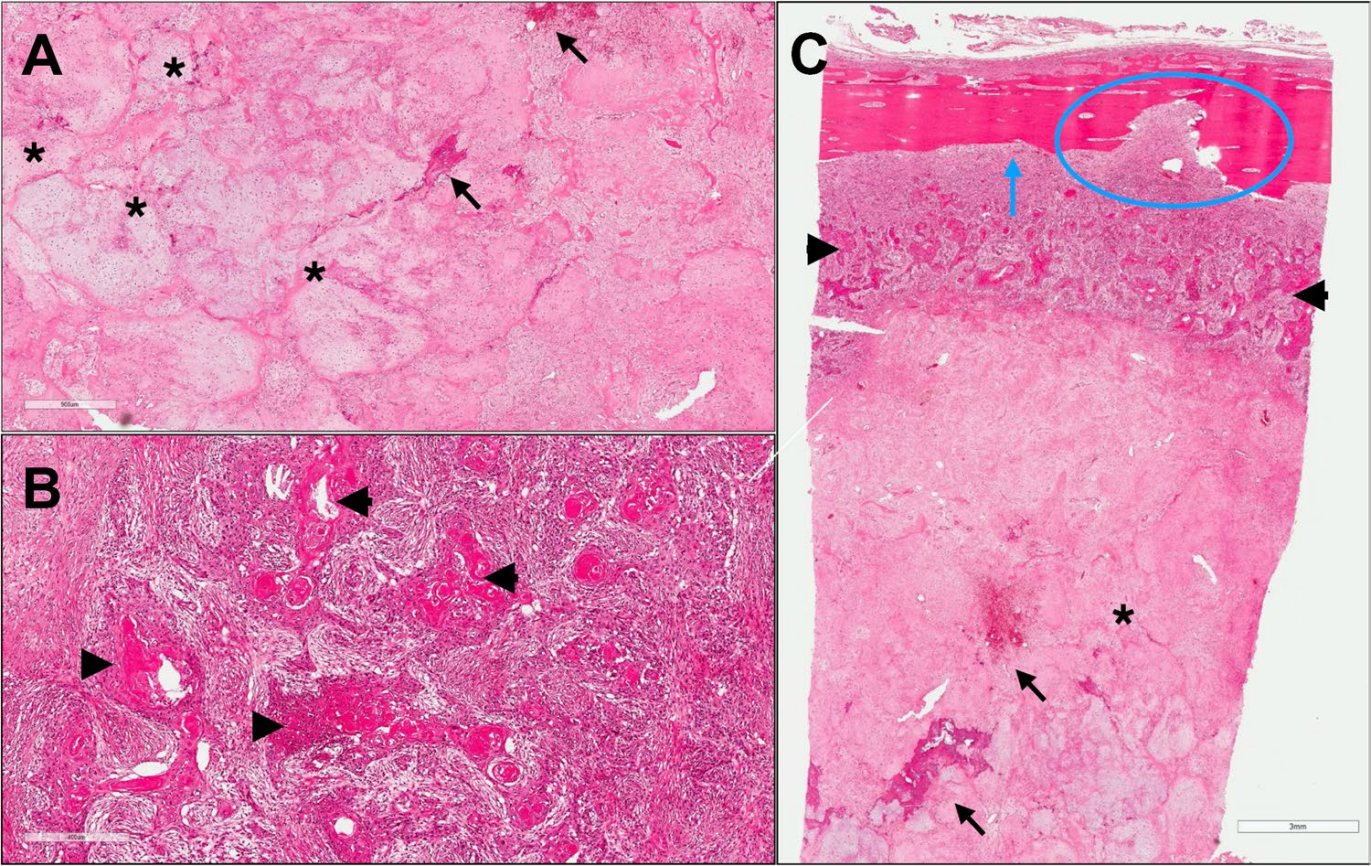
Moderate power field microscopy demonstrates [**A**—900 μm] multinodular growth (*) and larger nodular (black→) islands of hyaline cartilage (*) consistent with enchondroma; [**B**—400 μm] irregular islands of keratin forming squamous cells (➤)—with cellular pleomorphism visible on high power; and [**C**—3 mm] subcortical malignant squamous cell infiltration (➤) causing both shallow (blue →) and focal deep (blue circle) endosteal scalloping

Macroscopic appearances were substantiated on microscopy H&E assessment [Fig. 5] with multiple nodules of hyaline cartilage were seen abutting host bone, surrounded by a halo of reactive new bone (benign bone encasement growth pattern). There was no destructive or permeative growth with lack of trabecular entrapment by the enchondroma. Within the chondroid matrix was a mildly cellular population of bland chondrocytes in lacunar spaces, without significant myxoid change, atypia or mitotic activity [Fig. 5B]. Juxtaposed were irregular islands of keratin-forming and mitotically active pleomorphic squamous cells representing metastatic SCC [Fig. 5A]. There was also an area of infiltration along the endosteal interface with focal partial thickness erosion through the cortex by the SCC [Fig. 5C].

## Discussion

Tumour-to-tumour metastases (TTM) are an exceptionally rare occurrence, with first observations emerging in the early twentieth century [14, 15], the reported incidence exists in the order of 1:600 of metastatic disease [16]. Lung and breast cancers however are the most common donor metastases, with meningioma and RCC being the most common benign and malignant recipient tumours respectively [3, 17–23].

Whilst the literature contains very few instances of metastases to cartilage lesions [24, 25], we are expanding this small body of evidence with a case involving a head and neck squamous cell carcinoma within a solitary enchondroma.

In instances where malignancy spreads to benign lesions such as meningiomas, peripheral nerve sheath tumours, enchondromas or solitary fibrous tumours, it can masquerade as malignant transformation [2, 24, 26–28], or resemble intralesional dedifferentiation from a lower-grade variant such as chondrosarcoma [25, 29] or papillary thyroid carcinoma [30, 31].

Whilst TTM are infrequent, they are not insignificant as many become clinically apparent and hence identified prospectively. Many other intracranial [17] and systemic [30] TTM are only diagnosed in retrospect through post-mortem, however, and autopsy reports often highlight the substantial underappreciation for systemic dissemination of microscopic metastatic disease.

Although distinct pathological entities can occupy and coexist at the same anatomical location, we are reminded there is a clear technical and pathological delineation between the phenomena of tumour colocation/composition, collision, and TTM. Whilst different terminologies and classification systems have attempted to categorise these different entities [11, 32, 33], there are several definitions that remain consistent through the literature.

### Colocation/Composition

Colocated tumours are characterised by the coexistence of histologically distinct elements within a single mass. Such tumours occur with relative frequency in endocrine-related organs—including the kidneys [34], adrenals [35–37], and ovaries [38–40]—where an admixture of different epithelial components, such as germ cell and sex-cord stromal tumours, may develop independently of multiphenotypic differentiation. In these instances, the smaller neoplasm is typically distinguishable as a ‘nested’ entity within the host mass or situated ‘back-to-back’ along its periphery.

### Collision

Two independent primary neoplasms develop and coexist in the same location such as the skin, kidney, colon, stomach, bladder and skeleton [35, 41–47]. There is progressive incursion of the two lesions with progressive blending and histological admixture with imperceptibility of the interlesional interface.

Such examples are common with cutaneous malignancies where coexistence of separate but nearby carcinomas and melanomas can lead to incursion of both tumours into a shared location [48]. Reports include the viscera and musculoskeletal system where there is encroachment and progressive extension of a more aggressive (usually malignant) lesion into a benign pre-existing lesion. Such occurrences include osteosarcoma into fibrous cortical defect [49, 50] and enchondroma [51], synchronous cutaneous carcinomas and melanomas [48], cutaneous carcinoma and meningioma [53], and pulmonary squamous cell carcinoma with glandular papilloma and glomus tumour [53].

### Tumour-to-Tumour Metastasis

This term specifically describes the metastasis of one distant malignancy to another separate and histologically distinct tumour without direct extension [54]. Whilst improper utilisation of the ‘collision tumour’ continues to be applied to TTM, historical definitions included specific criteria to clearly delineate the difference between the two similar although separate entities.

Unlike collision metastases—such as the simultaneous coexistence of transitional cell and prostatic adenocarcinoma within regional lymph nodes [55–57]—a true tumour-to-tumour metastasis must satisfy the criteria established by Campbell [1968] [11]. This requires the presence of more than one distinct neoplasm, where the recipient is a true benign or malignant tumour and the donor is a confirmed metastasis; notably, this definition excludes collision tumours, intravascular tumour thrombus, and lymphatic metastases involving haematological malignancies.

Malignant myoepithelial carcinoma of bone could be considered within the differential diagnosis, with typical radiological features of a uniformly heterogeneous soft tissue mass with a permeative, moth-eaten appearance (100%) and cortical erosion (67%) often associated with an extraosseous soft tissue component [58]. However, its histological features typically differ from true chondroid matrix. Although intratumoural osseous metaplasia with calcification occurs in approximately 5% of cases and infiltrates of native trabecular bone may mimic intrinsic chondroid matrix, these remain uncommon features [58], and hence it was excluded pathologically.

Despite the literature comprising a significant and diverse catalogue of TTM [Table 1], this is the first reported case of a head and neck squamous cell carcinoma metastasising to an enchondroma.

**Table 1:** Review of tumour-to-tumour metastases in the literature

Primary tumour	Recipient tumour
Breast (carcinoma)	Ovary (mucinous) [59]
	Ovary (fibroma) [60]
	Ovary (teratoma) [61]
	Ovary (granulosa) [62]
	Adrenal (adenoma) [63]
	Phaeochromocytoma [64]
	Pleural (mesothelioma) [65]
	Bladder (SCC) [66]
	Meningioma [28]
	Pituitary [67]
	Chondrosarcoma [25]
	Renal (AML) [68]
	Renal (RCC) [11]
	Renal (carcinoid) [69]
NSCLC (unknown)	Meningioma [28]
	Renal (RCC) [75]
NSCLC (SCC)	Enchondroma [27]
	Enchondroma (Ollier) [24]
	Adrenal (adenoma) [31]
	Melanoma [70]
	Renal (oncocytoma) [74]
	Renal angiolipomyosarcoma [11]
	Renal (RCC) [11]
NSCLC (adenocarcinoma)	Testis (seminoma) [71]
	Renal AML [72]
	Renal (oncocytoma) [73]
	Chondrosarcoma (Ollier) [29]
	Renal (RCC) [11, 12, 16, 30]
	Meningioma [78]
	NSCLC (SCC) [30]
	Ileal (carcinoid) [16]
NSCLC (NET)	Renal (RCC) [76]
NSCLC (rhabdomyosarcoma)	Renal (RCC) [77]
SCLC	Thyroid (papillary) [30]
	Pancreas (adenoma) [30]
	Adrenal (adenoma) [79]
	Prostate (adenocarcinoma) [80]
	Renal (RCC) [11]
	Renal (oncocytoma) [81]
CRC	Renal (RCC) [16]
	Meningioma [28]
	Thyroid adenoma [82]
	Thyroid (Hurtle) [83]
	Thyroid (carcinoma) [31, 84, 85]
Oesophageal (SCC)	Gastric (adenocarcinoma) [86]
Appendiceal (adenocarcinoma)	Ovary (teratoma) [26]
Ileal (carcinoid)	Ovary (adenocarcinoma) [87]
Gastric (carcinoma)	
	Pituitary adenoma [89]
	Meningioma [17]
	Renal (RCC) [11, 90]
	Ovary (granulosa) [88]
Gastric (GIST)	Prostate (adenocarcinoma) [91]
Pancreatic (NET)	Renal (AML) [72]
	Thyroid (papillary) [30]
	Pituitary [92]
Pancreatic (adenocarcinoma)	Thymoma [17]
Cervical (adenocarcinoma)	Ovary (teratoma) [93]
	Renal (RCC) [19]
	Meningioma [94]
Endometrial (carcinoma)	Renal (RCC) [11]
Ovary (carcinoma)	Meningioma [95]
H&N (SCC)	Cervical LN Lymphoma [9, 13]
	Meningioma [8, 10]
	Renal RCC [11, 12]
Skin (scalp SCC)	Meningioma [52]
Skin (melanoma)	Renal (RCC) [16]
	Meningioma [96]
	Prostate (adenocarcinoma) [97]
Thyroid (carcinoma)	Renal (RCC) [11]
	NSCLC (adenocarcinoma) [98]
	PNST [2]
Prostate (adenocarcinoma)	Meningioma [28]
	Renal (RCC) [11, 55]
	Liposarcoma [99]
	Pituitary adenoma [92]
Renal (RCC)	Meningioma [28, 29]
	Thyroid (papillary) [30]
	Pancreatic (NET) [100]
	Glioblastoma [18]
	Phaeochromocytoma [101]
	Oesophageal (adenocarcinoma) [102]
Bladder (TCC)	Pleural (SFT) [19]
Leiomyosarcoma	Prostate (adenocarcinoma) [103]

*SCC* squamous cell carcinoma, *AML* angiomyolipoma, *RCC* renal cell carcinoma, *NSCLC* non-small-cell lung cancer, *NET* neuroendocrine tumour, *SCLC* small-cell lung cancer, *GIST* gastrointestinal stromal tumour, *LN* lymph node, *PNST* peripheral nerve sheath tumour, *SFT* solitary fibrous tumour

Whilst tumour-to-tumour metastasis remains a rare clinical entity, it remains a critical diagnostic consideration in oncologic imaging. In the presence of systemic metastatic disease, the identification of a biphasic morphology within a solitary lesion should prompt the radiologist to include TTM in the differential diagnosis. Maintaining this awareness is essential to ensure accurate staging prospectively, and to avoid the misinterpretation of complex secondary metastases or metachronous malignancy.

## Data Availability

Data availability is not applicable.
